# Mortality in non-elderly septic patients was increased with hypothermia and decreased with fever while mortality in elderly patients was not associated with body temperature: beware of some confounders!

**DOI:** 10.1186/s13054-020-03316-4

**Published:** 2020-10-13

**Authors:** Patrick M. Honore, Leonel Barreto Gutierrez, Luc Kugener, Sebastien Redant, Rachid Attou, Andrea Gallerani, David De Bels

**Affiliations:** ICU Department, Centre Hospitalier Universitaire Brugmann-Brugmann University Hospital, Place Van Gehuchtenplein, 4, 1020 Brussels, Belgium

We read with great interest the recent article by Shimazui et al. who concluded that in septic patients, mortality in non-elderly patients was increased with hypothermia and decreased with fever, while mortality in elderly patients was not associated with body temperature (BT) [1]. We would like to make some comments. As was alluded to by the authors themselves, BT measurement can be potentially confounded by various factors, including variation in the site of temperature measurement and whether or not patients receive antipyretics or targeted temperature management [1]. Nearly half of critically ill patients, especially those with septic shock, have or develop acute kidney injury (AKI) and 20–25% need renal replacement therapy (RRT) within the first week of their stay [2]. In the study of Shimazui et al., more than 60% of patients in both groups were in septic shock [1]. So, potentially, a third of patients may have had AKI, with 15% receiving continuous RRT (CRRT). CRRT is well known for blunting temperature in septic shock patients [3]. Moreover, in the elderly population, the risk of developing AKI is increased by age-related physiological changes, lower renal reserves, and multiple comorbidities that render them more susceptible to acute renal impairment [4]. In addition, elderly patients typically take more medications and undergo more procedures, which may endanger their renal function [4]. Hence, AKI is generally more common among the elderly [4]. A recent study found that frailty was a predictor for the development of AKI, increasing the likelihood by more than three times [5]. Therefore, it is a possibility that there were more patients with artificial hypothermia (below 36 °C) as a result of RRT in the elderly group of Shimazui et al., introducing a confounder to the study [1]. Unfortunately, data regarding AKI and RRT have not been reported in the study of Shimazui et al., so we are unable to confirm our hypothesis.

## Data Availability

Not applicable.

## Abbreviations

BT: Body temperature
AKI: Acute kidney injury
RRT: Renal replacement therapy
CRRT: Continuous renal replacement therapy
