# Supplementary material for: Mortality in non-elderly septic patients was increased with hypothermia and decreased with fever while mortality in elderly patients was not associated with body temperature: beware of some confounders!
Source: Crit Care. 2020 Oct 13;24:606. doi: 10.1186/s13054-020-03316-4 (PMC7552461; doi:10.1186/s13054-020-03316-4)
Supplement: Related texts

# Reply: Authors’ response

**Authors:** Takashi Shimazui, Taka-aki Nakada, Taku Oshima

**Affiliations:** grid.136304.3 0000 0004 0370 1101 Department of Emergency and Critical Care Medicine, Chiba University Graduate School of Medicine, 1-8-1 Inohana, Chuo, Chiba, 260-8677 Japan

We read with great interest the letter by Honore et al., concerning the potential confounding effect of continuous replacement therapy (CRRT) on body temperature (BT) measurement. The generally higher risk of developing acute kidney injury (AKI) in elderly patients might also influence the results.

To evaluate the impact of CRRT on our primary finding [1], we added analyses focused on AKI on admission and CRRT within the first 24 h from admission in the discovery cohort. According to the data availability, AKI was diagnosed based on the KDIGO criteria with some modification [6]: urine output in the first 24 h < 0.5 mL/kg, or serum creatinine levels on admission ≥ 0.3 mg/dL than baseline creatinine. Baseline creatinine was calculated using the Modification of Diet in Renal Disease (MDRD) study equation modified for the Japanese population [7], assuming an estimated glomerular filtration rate (eGFR) of 75 mL/min/1.73 m^2^ [6].

As Honore et al. have pointed out, the morbidity of AKI on admission was significantly higher in elderly patients compared to non-elderly patients (elderly 82.5%, non-elderly 73.8%, *P* = 0.0005 [chi-square test]). However, non-elderly patients were more frequently initiated on CRRT within first 24 h compared to elderly patients (elderly 17.2%, non-elderly 27.0%, *P* < 0.0001); non-elderly patients had higher chance of temperature alteration by CRRT. In addition, adding “CRRT” as covariates including previously reported analysis in the Cox regression for the hazard of death over 90 days by the BT adjusted with potential imbalances of age (per year), sex, chronic steroid use, and APACHE II score yielded results similar to the primary findings (non-elderly, BT < 36 °C and > 38.3 °C, *P* = 0.024 and *P* = 0.14, adjusted hazard ratio 1.70 and 0.73, 95% CI 1.07–2.71 and 0.48–1.10; elderly, BT < 36 °C and > 38.3 °C, *P* = 0.091 and *P* = 0.87, adjusted hazard ratio 0.62 and 0.96, 95% CI 0.36–1.08 and 0.63–1.47).

According to these results, we conclude that the BT, especially hypothermia, may be associated with mortality in non-elderly septic patients including those treated with CRRT, while the association between BT and mortality remains inconsistent in elderly septic patients even after adjusting the CRRT effects.
